# Seroprevalence of Visceral Leishmaniasis in Children up to 12 Years old Among Nomadic Tribes from Rural Areas of Pars Abad, Northwestern Iran: an Observational Study in 2015

**Published:** 2017-05-27

**Authors:** Hassan Ebrahimzade-Parikhani, Mehdi Mohebali, Zabiholah Zarei, Behnaz Akhoundi, Zahra Kakoei

**Affiliations:** 1 Department of Medical Parasitology and Mycology, School of Public Health, Tehran University of Medical Sciences, Tehran, Iran; 2 Center for Research of Endemic Parasites of Iran, Tehran University of Medical Sciences, Tehran, Iran

**Keywords:** Sero-prevalence, Visceral leishmaniasis, Nomadic tribes, Children, Iran

## Abstract

**Background:**

Since Pars Abad district had been known as a focus of visceral leishmaniasis (VL) in Ardabil Province but the prevalence of the disease in nomadic tribes has not been determined, thus, this study was conducted.

**Methods:**

This descriptive cross-sectional study was conducted on children up to 12yr old of nomadic tribes from Pars Abad County, Ardabil Province, Iran in 2015. For each individual, a questionnaire including age, sex, clinical manifestations, history of disease, and contact with reservoir hosts of VL were completed, separately. To determine VL seroprevalence, blood samples were collected from the children and after centrifugation, the plasma samples were tested using Direct Agglutination Test (DAT) for detection of anti-*Leishmania infantum* antibodies. Statistical analyses were performed using SPSS16.

**Results:**

From 776 children up to 12yr old, 2 (0.25%) showed anti-*L. infantum* antibodies at titers 1:1600 and only one case (0.13%) showed anti-*Leishmania* antibodies at titers 1:3200. The child with anti-*L. infantum* antibodies titers of 1:3200 showed mild fever for more than 2 months period, paleness, weakness and mild splenomegaly. After physical examination and confirmation of VL (kala-azar), the patient was treated with antileishmanial drugs.

**Conclusion:**

The findings indicated that *L. infantum* infection is being circulated with low prevalence in nomadic tribes of Pars Abad but it is necessary that the surveillance system is regularly monitored among physicians and public health managers in the studied areas.

## Introduction

Leishmaniasis has been reported from 98 countries in the world, in Iran this disease is endemic. More than 20 species of *Leishmania* produce diseases, and approximately 30 species of sand flies species (*Phlebotomus*) play a role in disease transmission (Alvar et al. 2012). The disease presents four clinical features, namely cutaneous leishmaniasis, mucocutaneous leishmaniasis (Espondia), visceral leishmaniasis (VL or kala-azar), and post kala-azar dermal leishmaniasis (PKDL) (Arevalo et al. 2007). Visceral leishmaniasis (VL), generally produced by *L. donovani* complex, is a dangerous form of leishmaniasis and transmitted by the bite of female sandflies (Mohebali 2013).

This disease is manifested by prolonged systemic symptoms such as fever, hepatomegaly, splenomegaly, weight loss, and pancytopenia (Alvar et al. 2012, Mohebali 2012). Visceral leishmaniasis is projected to about 500,000 cases of morbidity and 59,000 cases of mortality every year in the world (Werneck 2014). This disease has high Case Fatality Rate (CFR) in untreated patients, underweight children and cases of HIV/VL co-infection (Cavalcante and Vale, 2014). Visceral leishmaniasis is mostly reported in six countries: India, Ethiopia, Nepal, Sudan, Bangladesh, and Brazil (Boelaert et al. 2000). The hazard in gaining the illness is mediated through unfortunate housing environments, nonexistence of private protective processes against the vector and migration that leads to non-immune hosts entering VL-endemic regions (Bern et al. 2008). Kala-azar is endemic in many Asian countries and the Middle East. In Iran, VL is observed in sporadic and endemic forms, so in many parts of Iran the disease is sporadic and in some parts of the provinces such as Ardabil, East Azerbaijan, Bushehr and Fars, the disease is seen as endemic (Mohebali et al. 2011).

Remarkably, from the 7204 serum sample gathered from domestic dogs in rural communities well-known as the endemic foci of human VL in Iran, 879 (12.2%) were seropositive by titers of ≥1:320 (Mohebali et al. 2005, Mohebali et al. 2006, Moshfe et al. 2008, Moshfe et al. 2009). Also, in a study conducted by Moshfe et al in 2006–2007 on canine Visceral leishmaniasis in Meshkin Shahr District in northwestern Iran, 17.4% of serum sample were positive by DAT (1:320 and higher). Moreover, a quarter of seropositive dogs displayed clinical VL symptoms, and cachexia and alopecia were the highest clinical signs in the seropositive dogs (Mohebali et al. 2005).

Annually, approximately 100–300 new symptomatic cases of VL are registered in the Health Care System of Iran (Mohebali 2013). Out of the 31 provinces of Iran, more than 2000 cases of VL were reported up to 2012 (Mohebali 2012). In this report, 44.6% of the cases were from the northwestern region of Iran (Mohebali 2013). During the last decade in Iran, an annual average incidence rate of disease was reported as 0.449 per 100000 at-risk populations. Here, in the northwestern part of Iran, with an incidence rate of 57 cases per 100, 000, have the maximum incidence rate of the disease in Iran (Mohebali 2013).

In this study, the direct agglutination test (DAT) was used as a sero diagnostic tool due to its simplicity, cost-effectiveness, appropriate sensitivity (92–100%) and specificity (72–100%), valid test results and its suitability for use in field conditions (Elmahallawy et al. 2014). Since Ardabil Province is one of the foci of VL and the prevalence of the disease especially on nomadic tribes of this province has not been evaluated, this present study was conducted to determine the seroprevalence of VL in the nomadic tribes of Pars Abad County to rapid case finding, and to provide suggestions for the prevention of the disease among nomadic tribes in the study area.

## Materials and Methods

### Study area

This survey was carried out in the nomadic tribes of Pars Abad district from 21 March 2014 to 20 March 2015. Pars Abad district is located in the north of Ardabil Province, northwestern Iran. The study area has mild weather condition in the summer and cold in the winter with an average altitude of 32m above the sea level and a landmass of 1383 km^2^ (14 percent of the Ardabil Province area). Pars Abad area is found in the northern part of the Ardabil Province, located between the orbits of 39 degrees and 12min to 39 °C and 42min north latitude and 47 degrees and 10 min to 48 degrees and 21min east of the Greenwich meridian. Pars Abad area have 3 districts, 2 cities and 6 villages. According to the 2011 Iranian census, the population of this county was 88,924 (https://www.amar.org.ir).

### Blood sampling

This study was conducted on children ≤12yr old of nomadic tribes from rural areas of Pars Abad, northwestern Iran. Altogether, the blood samples were prepared from 776 children. For each that entered the study, questionnaires that included individual characteristics such as age, sex, location, clinical signs, history of the disease, and contact with dog were provided and filled. For serologic studies, blood samples were collected, and the plasma samples were separated by centrifugation and stored at −20 °C until their examinations.

### Performance of direct agglutination test (DAT)

The *L. infantum* antigens for this study were prepared in the Leishmaniasis Laboratory of the School of Public Health, Tehran University of Medical Sciences, Tehran, Iran. The principal phases of the procedure for preparing direct agglutination test antigen were mass production of promastigotes of Iranian strain of *L. infantum* [MCAN/IR/07/Mohebgh. (GenBank accession no. FJ555210)] in RPMI1640 medium (Biosera, South America), plus 10% fetal bovine serum (Biosera, South America), followed by tripsinization of the parasites, staining with Coomassie brilliant blue R-250 (Sigma, USA) and fixing with formaldehyde 1.2% (El Harith et al. 1988, Zijlstra et al. 1991, Mohebali et al. 2006).

All the collected plasma samples were tested by DAT. The titration of *Leishmania-*specific antibodies was performed by following the general procedures described by Mohebali et al. (2006). Primarily, two dilutions of 1:800 and 1:3200 were made and tested for screening. The samples that were positive with the titer of 1:800 were diluted up to 1:102400 in a V-shaped microtiter plate into a dilution fluid containing 0.9% saline and 0.78% 2-mercaptoethanol. One equal volume (50μl) of antigen suspension was added to each well. The results were read after 18–24 h incubation in a wet room at room temperature. The highest dilution at which agglutination was still visible in comparison with positive and negative controls titer was defined as the titer of the sample. Compact blue dots were scored as negative and large diffused blue mats as positive.

The characteristics of seropositive cases of the disease were reported to the health centers of Pars Abad District to receive suitable cure, if necessary. Antigen control well (antigen and diluent plasma only) and the known negative and positive controls were tested in each plate daily. Based on prior studies (El Harith et al. 1988, Mohebali et al. 2006), titers of ≥ 1:3200 were considered as seropositive, and persons with suspected (a titer of 1:1600) or lysed samples were re-sampled 2 to 3 weeks later (Schallig et al. 2001, Mohebali et al. 2006, Kakooei et al. 2014).

### Ethical considerations

Informed written consent was obtained from the parents of the children examined. This study was approved by the Research Ethical Review Committee of Tehran University of Medical Sciences, Tehran, Iran.

### Statistical analysis

Chi-squared and Fisher exact tests were used for the assessment of the relation between two quantitative variables (sero-prevalence values in relation to sex and age group). Statistical significance was assumed if P< 0.050. All reported P values are two-sided. Statistical analyses were performed using SPSS software (version 16, SPSS Inc., Chicago, IL, USA).

## Results

Altogether, 776 blood samples were collected from the children up to 12 years old from nomadic tribes of north of Ardabil Province. Four hundred and three of them (51.9%) were male, and 373 (48.1%) were female. The sex ratio (male/female) of the studied population was 1.08. From the 776 children, 3 (0.3%) showed anti-*Leishmania* antibodies at titers ≥ 1:1600, and from these only one case showed anti-*L. infantum* antibodies at titers of 1:3200 (Table 1). Hence, the prevalence of the disease (Titers of 1:3200) in the studied areas was estimated as 1.28 per 1000 at-risk populations. Furthermore, the seropositive case presenting anti-*Leishmania* antibodies at titers 1:3200 was a 10 year old female from AGH Ghabagh (Hosein Gheshlagh) village with a positive history of the disease with remittent fever, mild splenomegaly and cutaneous scar in the face in two or three months prior to her disease diagnosis. This patient was hospitalized in the pediatric hospital and was successfully treated with Glucantime® as the first line of anti-leishmanial drugs.

**Table 1.** Seroprevalence of human visceral *Leishmania infantum* infection by gender in children up to 13 years old of nomadic tribes of Pars Abad County from Ardabil Province, Iran in 2015

**Anti-*Leishmania infantum* antibody titers**					

**Gender**	**Number of examined**	**Negative (%)**	**1:800**	**1:1600**	≥**1:3200**

			**Number (%)**	**Number (%)**	**Number (%)**
**Male**	403	403 (100%)	0 (0%)	0 (0%)	0 (0%)
**Female**	373	370 (99.2%)	0 (0%)	2 (0.5%)	1 (0.3%)
**Total**	776	773 (99.6%)	0 (0%)	2 (0.3%)	1 (0.1%)

The relation between human *Leishmania* infection titers and sex and age group was not statistically significant, with P-values of (P= 0.197) and P= 0.332), respectively probably because of the low number of seropositivities. The frequency of anti-*Leishmania* antibody titers with DAT according to the age groups is shown in Table 2. Age group and sex distribution of samples is presented in Table 3. In Table 3, the age group, sex, location and anti*-Leishmania* antibody titers of 3 seropositive cases of VL has been showed.

**Table 2.** Seroprevalence of human *Leishmania infantum* infection by age group in nomadic tribes of Pars Abad County from Ardabil Province, Iran in 2015

**Anti-*Leishmania infantum* antibody titers**					

**Age group (yr)**	**Number of examined**	**Negative (%)**	**1:800**	**1:1600**	**1:3200**

			**Number (%)**	**Number (%)**	**Number (%)**
**<4**	202	201 (99.5%)	0 (0%)	1 (0.5%)	0 (0%)
**5–8**	280	280 (100)	0 (0%)	0 (0%)	0 (0%)
**9–12**	294	292 (99%)	0 (0%)	1 (0.5%)	1 (0.5%)
**Total**	776	773 (99.6%)	0 (0%)	2 (0.3%)	1 (0.1%)

**Table 3.** Anti-*Leishmania infantum* antibody titers of three seropositive cases of visceral *Leishmania infantum* infection by direct agglutination test with respect to their age, gender and locality in nomadic tribes of Pars Abad from Ardabil Province, Iran 2015

**Case No.**	**Age (yr)**	**Gender**	**Location**	**Tribe**	**Antibody Titer**
**1**	9	Female	Pars Abad	Asad Kandi	1:1600
**2**	≤1	Female	Pars Abad	Griloo	1:1600
**3**	10	Female	Pars Abad	Hossin Gheshlagh	1:3200^*^

* This patient had showed clinical signs and symptoms

**Fig. 1.**
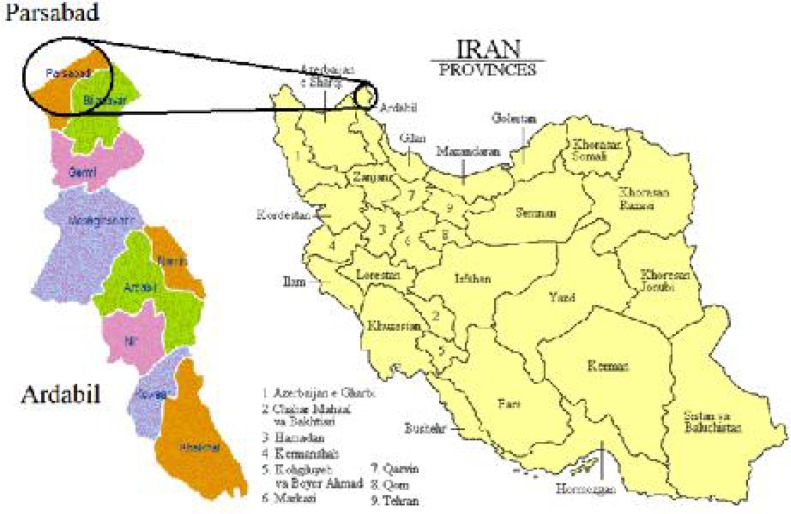
Situation of Ardabil province in Iran and location of study areas in Ardabil Province (Pars Abad)

## Discussion

This study was conducted to assess the epidemiological characteristics of VL in Pars Abad in the north of Ardabil Province (northwestern Iran). The prevalence of visceral *L. infantum* infection in the Pars Abad district was 1.28 per 1000 at-risk populations. Females and those belonging to the age group of 9–12 years old are at higher risk of disease, in comparison with the other groups.

In recent years, various and scattered studies about several aspects of VL have been conducted in Ardabil Province in northwestern Iran. The results showed that VL is endemic in some areas of the province, and most of cases of VL were reported from Meshkin Shahr District (Soleimanzadeh et al. 1993, Edrissian et al. 1996, Mohebali et al. 2006).

In this study, we observed that from the 776 persons that entered the study, 3 (0.3%) of them showed anti-*L. infantum* antibodies at titers ≥ 1:1600, and from these only one case (0.1%) showed anti-*Leishmania* antibodies at titers ≥ 1:3200. In a sero-epidemiological study that was conducted by in our study, only one seropositive case of the disease with titers of ≥1:3200 was diagnosed, which included a female in the age group of 9–12yr old. In a study performed by Abbaszadeh-Afshar et al. (2015) 0.50% of 6 seropositive cases were males and 0.50% were female, and children belonging to the age group of 5–8yr old showed the highest seroprevalence rate (4.1%) of the disease, A similar result was found by Mahmoudvand et al. (2011) in which 41.66% (5 cases) of 14 seropositive cases were females and 58.34% (9 cases) were males.

## Conclusion

*Leishmania infantum* infection is being circulated with low prevalence in the nomadic tribes of Pars Abad area, however, it is necessary for the surveillance system to regularly monitor the infection in the studied areas. Appropriate counseling should be provided to military personnel, researchers, and other groups of travelers who may be exposed to sand flies in the endemic areas. Early case detection and treatment can reduce the impact of severe illness and death. Further studies on domestic dogs as principal animal reservoir host of VL in the nomadic tribes of Pars Abad are recommended.
